# Delimitation of Endangered *Telmatobius* Species (Anura: Telmatobiidae) of the Chilean Salt Puna

**DOI:** 10.3390/ani14243612

**Published:** 2024-12-15

**Authors:** Pablo Fibla, Paola A. Sáez, Gabriel Lobos, Nicolás Rebolledo, David Véliz, Luis Pastenes, Talía del Pozo, Marco A. Méndez

**Affiliations:** 1 Departamento de Ciencias Ecológicas, Facultad de Ciencias, Universidad de Chile, Las Palmeras 3425, Ñuñoa, Santiago 7800003, Chile; paolasaezg@gmail.com (P.A.S.); dveliz@uchile.cl (D.V.); 2 Centro de Ecología Aplicada (CEA), Príncipe de Gales 6465, La Reina, Santiago 7850298, Chile; 3 Centro de Gestión Ambiental y Biodiversidad, Facultad de Ciencias Veterinarias y Pecuarias, Universidad de Chile, Avenida Santa Rosa 11735, La Pintana, Santiago 8820000, Chile; galobos@yahoo.com; 4 Ecodiversidad Consultores, Pasaje Riñihue 1022, Puente Alto, Santiago 8150000, Chile; nico.rebolledo.f@gmail.com; 5 Departamento de Biología y Química, Facultad de Ciencias Básicas, Universidad Católica del Maule, Avenida San Miguel 3605, Talca 3480112, Chile; lpastenes@ucm.cl; 6 Núcleo de Investigación en One Health (NIOH), Facultad de Medicina Veterinaria y Agronomía, Universidad de Las Américas, Santiago 7500975, Chile; tdelpozo@udla.cl; 7 Center of Applied Ecology and Sustainability (CAPES), Libertador Bernardo O’Higgins 340, Santiago 7510177, Chile; 8 Cape Horn International Center (CHIC), O’Higgins 310, Cabo de Hornos 6200000, Chile; 9 Laboratorio Natural Desierto de Atacama (LANDATA), Avenida Angamos 0610, Antofagasta 1240000, Chile

**Keywords:** species delimitation, taxonomy, amphibians, Andean Altiplano, Tauca paleolake

## Abstract

Clarifying the taxonomic status and distribution of endangered species is crucial to their conservation. In this study, we contrasted different lines of evidence (morphology, mtDNA, and nucDNA: microsatellites and SNP) to clarify the taxonomic status of endangered *Telmatobius* water frog species and unidentified populations that inhabit the Salt Puna in Chile. We studied population differentiation and species divergence by performing morphometric, population genetic and species delimitation analyses. The results confirmed the species status of *Telmatobius fronteriensis* and *T. philippii*, as they exhibited morphometric, mitochondrial and genomic SNP divergence. Although Bayes factor delimitation analysis indicated that the *Telmatobius* populations of Ascotán and Carcote could represent a new species, their few mitochondrial differences and similar morphology with respect to *T. philippii* suggested otherwise. Instead, they can be considered an evolutionarily significant unit of *T. philippii* that has differentiated from the type locality. These results extend the geographic distribution of *T. philippii*, which is categorized as critically endangered by the IUCN.

## Simple Summary

Understanding the taxonomic status and distribution of endangered species is very important for their conservation efforts. We used morphology, mitochondrial, and nuclear DNA (microsatellites and SNPs) to evaluate the actual classification (taxonomy) of a group of endangered Andean water frog species (genus *Telmatobius*) and localities whose identity has been unclear. Both morphological and molecular analyses agree with the taxonomy of the focal species and suggest that populations with an unclear status belong to one of the studied species, *Telmatobius philippi*, which was previously restricted to two localities. This range extension may contribute to clarifying the conservation status of this species.

## 1. Introduction

Species delimitation, the process of determining which groups of individual organisms constitute populations of a single species and which constitute different species [1], is an important step towards the protection of poorly known organisms as species are considered the fundamental unit in biological conservation [2,3,4,5,6]. The development of genome-scale analyses now permits the detection of fine-scale patterns of genetic variation [7,8,9,10]. For instance, phylogenomic analyses can robustly recover true phylogeographical lineages compared to conventional phylogenies by incorporating far more polymorphic sites [11]. Although this improves our capacity to delineate phylogeographic lineages and discover new species [12,13,14], it implies a risk of taxonomic over-splitting because intraspecific variation (population structure) can be mistaken for (or considered) interspecific divergence [15,16,17]. Therefore, when delimiting species, it has been recommended to contrast multiple methods and characteristics beyond genomic data (i.e., integrative taxonomy) conservatively [2,9].

The Andean zone of northern Chile (dry Central Andes) is embedded in the South American Arid Diagonal [18] and forms an important part of the Atacama Desert, one of the most arid places on the Earth. The main geological and altitudinal divisions of this zone are the western Andean flank (and slope) and the Altiplano [19], or Andean plateau, which reaches an average altitude of 4000 m.a.s.l. [20]. The bodies of water in the Chilean Altiplano are often geographically isolated without hydrological connections and separated by long distances [21]. From a hydrological point of view, this region is fragmented into a series of closed drainage basins that are partially or totally isolated from each other and delimited by irregular relief, mountain ranges and even volcanoes [22]. The high degree of geographic isolation of the wetlands present in the Altiplano of northern Chile and the presence of geomorphological barriers between the hydrographic basins would have stimulated the origin of a high number of aquatic endemism in this region, especially in systematic groups such as amphibians and fishes as well as aquatic invertebrates [21,23,24,25].

One of the most diverse yet poorly known groups of frogs in high-altitude, tropical Andean environments is the genus *Telmatobius* Wiegmann, 1834. Its distribution at high elevations and fully aquatic habits are the most remarkable features of these amphibians. The taxonomy and systematics of the genus *Telmatobius* are considered complex because of different factors that preclude a clear delimitation of its species, including contrasting patterns of intra- and interspecific morphological variation [26,27,28] and the existence of cryptic lineages [29]. In Chile, there are seven recognized *Telmatobius* species [30,31]. These species inhabit Andean freshwater systems near or within the Atacama Desert area [32,33,34,35].

The *Telmatobius hintoni* group include four Altiplanic species distributed in Bolivia, *T. hintoni* and *Telmatobius huayra*, and Chile, *Telmatobius fronteriensis* and *Telmatobius philippii*. The Chilean populations of this group inhabit thermal springs located in the closed hydrographic basins of the Chilean Altiplano in an area also known as “Salt Puna” (because of the presence of numerous salt flats). Although the taxonomic status of these Chilean *Telmatobius* populations (and species) has been questioned because of the phenotypic similitude and low genetic divergence observed between them [25], there are no integrative studies on the taxonomic status of *T. fronteriensis* and *T. philippii* or of the many unidentified surrounding *Telmatobius* localities whose taxonomic status is unclear in the Ascotán and Carcote Salt Pans [36,37,38].

**Figure 1:**
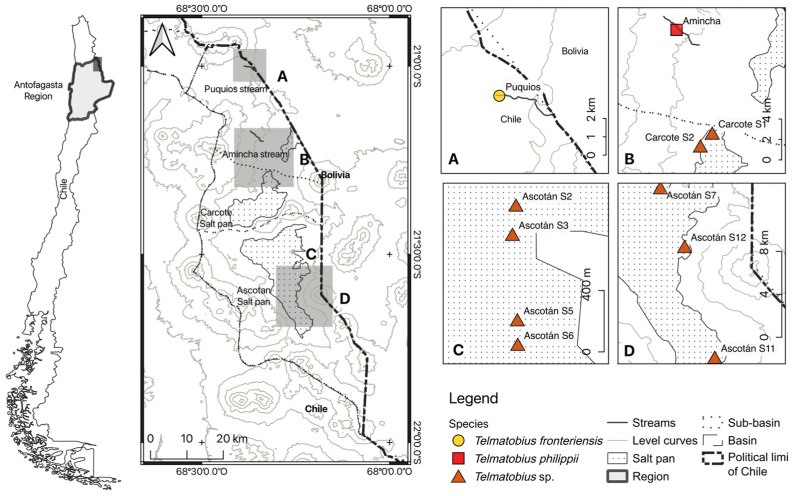
Study area. Map indicating *Telmatobius* sampled localities (sites) in the Chilean Salt Puna. The circle in A and the square in B indicate the type localities of *T. fronteriensis* and *T. philippii*, respectively, whereas the unidentified localities (triangles in B, C and D) correspond to *Telmatobius* sp. Ascotán spring (S) names follow [40]. Carcote S2 correspond to “Aguas Calientes” spring in [37].

It has been described that the existence of a temporal gap between the taxonomic knowledge of Chilean *Telmatobius* populations and their anthropic threats (i.e., threats are known before their taxonomic identity) may be a potential risk factor for the conservation of these endemic amphibians [36]. Many unidentified *Telmatobius* localities, such as the springs of the Ascotán and Carcote Salt Pans, are located in an area in which accelerated industrial growth and mining activities (also known as the “Lithium Triangle” of South America) threaten several species [39]. *Telmatobius fronteriensis* and *T. philippii*, which also inhabit this area, share the highest priority for conservation among the Chilean amphibian species [31], so it is important to clarify the status of these species and unidentified localities. Therefore, we contrasted different lines of evidence (morphology, mtDNA, and nucDNA: microsatellites and SNP) to study population differentiation and species divergence and clarify the taxonomic status of these endangered *Telmatobius* water frog species and localities that inhabit the Salt Puna in Chile. We used mitochondrial and microsatellite data to first evaluate if the type locality of *T. philippii*, Amincha, and several nearby localities in the Salt Pans of Ascotán and Carcote (Figure 1) that had been considered that *T. philippii* [36] constitute a unique panmictic unit or differentiated units; it has been suggested that the *Telmatobius* locality “Aguas Calientes” in Carcote corresponds to *T. halli* [37], but this hypothesis was rejected by [38]. Then, we applied morphometric, phylogenetic and species delimitation analyses to examine the species boundaries of the Chilean species (including unidentified localities) belonging to the *Telmatobius hintoni* group [25].

## 2. Materials and Methods

### 2.1. Sampling

In 2016, 2017, and 2022, biological samples of *Telmatobius* specimens (larvae, juveniles, and adults) were obtained from locations within the study area (Figure 1). Frogs were captured under vegetation and among the springs’ rocks using fishing nets in each locality. After capture, specimens were anaesthetized using benzocaine (ethyl 4-aminobenzoate; 300 mg/L) diluted in water obtained from each locality of capture and then released at the same collection site immediately after full recovery from anaesthesia. A small piece (vol. < 0.1 mL) of the interdigital membrane (adults and juveniles) or tail membrane (larvae) was used as the DNA source. Tissue samples were stored in absolute ethanol. The sample sizes by locality and marker are shown in Table 1. Table S1 shows the sample sizes by life stage. Differences in sample sizes between markers and localities are related to the requirements and focus of each analysis and also reflect differences in specimen abundance during field campaigns.

### 2.2. Lab Procedures and Population Genetic Analyses

In this analysis, we included the *T. philippii* type locality, Amincha, and the nearby *Telmatobius* localities, within the Ascotán and Carcote Salt Pans, that also has been considered *T. philippii* (Table 1). Total DNA was isolated from *Telmatobius* samples using the salt extraction method (modified from [41]). The integrity of the extracted DNA was verified by DNA agarose gel electrophoresis (2%) with GelRed™ staining (Biotium, Fremont, CA, USA) and visualized with a UV transilluminator. DNA quantity and quality assessments were performed using a NanoDrop Lite™ spectrophotometer (Thermo Fisher Scientific, Waltham, MA, USA).

#### 2.2.1. Mitochondrial Control Region and Microsatellite Data

A fragment (±920 bp) of the mitochondrial control region (CR) and microsatellite (SSR) markers were amplified for population genetic analyses. The primers used to amplify (PCR) the CR fragment were Tchu_cytb(1103) (forward, CAA CAA TCG GAG CAC TAG A) described by [42] and Tchu_dloop(2343) (reverse, CCT TGC TCC TGA CTT CTT). The amplification mixture (PCR) consisted of 3.0 mM MgCl_2_, 0.4 mM dNTPs, 0.15 mM of each primer, 1.0 U of Taq (Invitrogen™, Waltham, MA, USA), and 60 ng of total DNA in a final volume of 30 μL. The thermal profile was 3 min at 94 °C for the initial denaturation; 40 cycles of 30 s at 94 °C, 45 s at 54–58 °C for annealing, and 80 s at 72 °C for extension; and the final extension for 10 min at 72 °C. The obtained sequences were manually edited and aligned (using the ClustalW algorithm) using BioEdit v.7.2.0 [43]. The DNA sequence matrix used in the analyses was built using the ClustalW algorithm for multiple alignments integrated within BioEdit v.7.2.0.

In the case of microsatellite (SSR) markers, eight polymorphic loci (Table S2) were selected and amplified as described by [44]. The genotypes of the microsatellite loci were manually recorded using GeneMarker software version 1.91 [45]. All loci within each locality were checked for the presence of genotyping errors, null alleles, significant allele dropout, and stuttering with Micro-Checker v.2.2.3 [46] using Bonferroni-adjusted 95% confidence intervals (Dunn–Sidak) derived from 10,000 Monte Carlo simulations. Linkage disequilibrium (LD) between pairs of loci was calculated with GENEPOP v.4.4.2 [47] using the Markov chain method (10,000 dememorization steps, 1000 batches, and 5000 iterations/batch) and a sequential Bonferroni correction. Table 1 presents the number of samples (by locality) used in mitochondrial and microsatellite analyses.

#### 2.2.2. Statistical Analyses

To evaluate population differentiation, we inferred a network of haplotypes by applying the median-joining algorithm [48] implemented in PopART (http://popart.otago.ac.nz/index.shtml accessed on 8 April 2023; [49]) in the CR data, and we conducted principal component analysis (PCA) on the SSR data using the Adegenet package v.2.1.10 [50] implemented in R software v.4.2.2 [51]. Pairwise F_ST_ among localities were estimated in Arlequin v.3.5 [52] for CR data and in GENETIX v.4.05.2 [53] for microsatellite data. Statistical significance was assessed using 10,000 permutations.

We also explored genetic diversity per locality. The number of haplotypes and polymorphic sites, pairwise differences and nucleotide diversities were estimated with DnaSP v.5.0 [54] using CR data. Observed and expected heterocigocities, the mean number of alleles per locus and inbreeding coefficients (F_IS_) per locality were estimated on microsatellite data using GENETIX v.4.05.2. F_IS_ statistical significance was assessed using 10,000 permutations.

### 2.3. Phylogenetic, Species Delimitation, and Morphometric Analyses

In this case, we included samples from the species *T. fronteriensis*, *T. philippii*, and *T. huayra* and the *Telmatobius* localities of Ascotán and Carcote. Sample sizes are shown in Table 1. In species delimitation analyses, we sampled just a few individuals per basin, considering the suggestions of [55,56] when performing Bayes factor and PTP delimitation analyses.

#### 2.3.1. Cytochrome-b Data

A mitochondrial cytochrome-b (Cytb) fragment was used in the phylogenetic and species delimitation analyses with mitochondrial data. To construct the DNA matrix, partial sequences of Cytb (±940 bp) of the different species were amplified with the same pairs of primers and PCR conditions used by [25]. In the case of *T. huayra*, we used a Cytb sequence (GU060597.1) from the Genbank repository [28].

#### 2.3.2. Sequencing and SNP Calling

For each sample (Table 1), DNA was extracted and subjected to massively parallel sequencing at Dart Diversity Arrays Technology Pty Ltd. (DArT; Canberra, Australia). DNA was digested with the restriction enzymes *Sbfl* and *Pstl* as described by [57]. Fragments larger than 200 bp were ligated with an eight-base pair barcode and amplified by PCR. The PCR products were standardized and sequenced on the HiSeq 2500 System (Illumina Inc., San Diego, CA, USA).

Dart Diversity’s bioinformatics service performed the demultiplexing and removal of the DNA barcodes. More information about the detection of SNPs is described by [57,58]. Raw SNP data from Dart (103,269 SNPs, missing data = 26.46%) were filtered using the dartR package v.2.7.2 [59] implemented in the R software (R core Team). To improve the quality of our dataset and reduce genotyping errors, we retained only one SNP in the reads containing two or more SNPs. We eliminated loci as follows: (i) loci with a read depth lower than 5 or higher than 200, (ii) loci with <99% reproducibility, (iii) monomorphic loci, (iv) loci with >10% missing data, (v) individuals with >5% missing data, and (vi) all SNPs with a minimum allele frequency <1%. We also eliminated all loci identified as under selection using three different approaches: (i) the method based on likelihood implemented in the outflank function of the dartR package, (ii) the Bayesian method implemented in the BayeScan program v.2.1 [60], and (iii) the method based on the relationship between F_ST_ and the heterozygosity of the Fsthet library [61] implemented in R software. One of the loci pairs exhibiting LD > 0.9 in all sampling sites was filtered with PLINK 1.9 software [62]. Finally, the SNP dataset consisted of 21 genotypes and 1226 unlinked SNP (0.47% missing data).

#### 2.3.3. Phylogenetic and Species Delimitation Analyses

We applied the *gl2fasta* function (method 3), which concatenates SNP bases across loci and generates an FASTA sequence alignment then used in the phylogenetic analysis. The standard ambiguity codes replaced heterozygous positions. Principal coordinate analysis (PCoA) with SNP data was used to visualize species divergence in a multivariate space. Both functions, namely PCoA and concatenate alignment, are implemented in the dartR package.

Maximum likelihood (ML) phylogenetic analyses were conducted in RAxML v.8.2.1 [63] using separately the Cytb and (FASTA) SNP matrices. The GTRCATX model (estimated bases and four gamma categories) was applied to both datasets as suggested by ModelTest-NG (AICc score = 10,474.9558; [64]). Statistical node support was evaluated using 5000 bootstrap replicates (i.e., 5000 distinct ML trees starting from 5000 distinct randomized maximum parsimony starting trees) and 2000 bootstrap replicates in mitochondrial and SNP datasets, respectively.

A Bayesian PTP maximum likelihood (bPTP-ML; [56]) species delimitation analysis was carried out in the web server (https://species.h-its.org/ accessed on 10 September 2024) using the ML tree inferred using Cytb data. For this analysis, we considered 500,000 MCMC generations, with a thinning of 100 and 10% of burnin.

Bayes factor delimitation (BFD) implemented in BEAST v.2.6.6 [65] was performed to contrast, in terms of their marginal likelihood, three different species delimitation hypotheses based on SNP data: (i) the “current taxonomy hypothesis”, which considers three species (*T. huayra*, *T. fronteriensis*, and *T. philippii*); (ii) the “splitter hypothesis”, which considers the aforementioned species and a fourth, new species constituted by *Telmatobius* species of Ascotán and Carcote; and (iii) the “lumper hypothesis”, which considers *T. huayra* as a species and the remaining localities as another single species. The marginal likelihoods of the species delimitation hypotheses were estimated by conducting a path sampling analysis using 20 steps (100,000 Markov chain Monte Carlo steps [10% burnin] and 10,000 pre-burnin steps) on each model. The convergence of the analyses was evaluated in terms of their effective sample size (>200). To compare the strength of the support from Bayes factor (BF) comparisons of competing models, we applied the approximation of [66]. A positive BF statistic (2 × log*e*) reflects evidence favouring model 1, whereas negative BF values are considered evidence favouring model 2. The following ranges were used to categorize the BF statistical support: 0 < 2 × log*e*BF < 2 was not worth more than a bare mention, 2 < 2 × log*e*BF < 6 was positive evidence, 6 < 2 × log*e*BF < 10 was strong support, and 2 × log*e*BF > 10 was decisive support.

#### 2.3.4. Morphometric Analysis

To compare adult specimens of all the studied species (including the type specimen of *T. huayra* and topotypic material of *T. fronteriensis* and *T. philippii*), we performed linear morphometric analyses using preserved specimens deposited in the DBGUCH collection of the University of Chile and in the Bolivian Collection of Fauna (CBF). The collection codes of the measured specimens are detailed in Appendix A. Eleven external variables were measured on each specimen: snout–vent length (SVL), head width (HW), head length (HLt), internostril distance (IND), eye–snout distance (ES), the distance between anterior (medial) eye commissures (EAD), the distance between posterior (lateral) eye commissures (EPD), femur length (FmL), tibia length (TbL), tarsus length (TrL), and the length of the fourth toe (T4L). All bilateral traits were measured from the right side. Measurements were obtained using a digital Vernier (precision = 0.01 mm). Morphological variation between species/localities was represented using PCA on log-transformed morphometric variables. Significant differences were assessed by PERMANOVA (based on Euclidean distances matrix) with 9999 permutations, followed by post hoc pairwise comparisons using the Hotelling–Lawley statistic. The statistical significance of pairwise comparisons was adjusted by Bonferroni’s correction. Morphometric analyses were conducted using MASS [67], Vegan [68] and RVAideMemoire [69] packages in the R environment. The package ggplot2 [70] was used to create scatter plots for PCA results.

## 3. Results

### 3.1. Population Genetic Analyses

**Figure 2:**
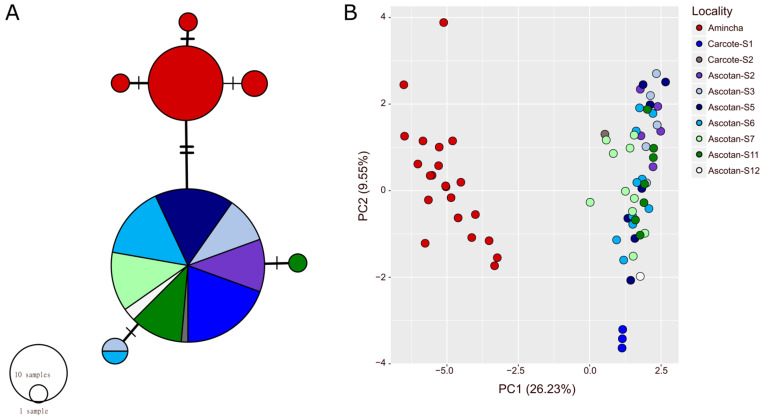
Results of the population genetic analyses. Median-joining network inferred using the amplified fragment of the mitochondrial CR (**A**) and microsatellite PCA scatter plot presenting the two first PCs (**B**). The percentage of genetic variance explained by each PCA component is indicated between parentheses.

The mitochondrial CR median-joining network (Figure 2A) was composed of seven haplotypes separated into two haplogroups: one haplogroup contained individuals from the Amincha locality and the second haplogroup included individuals from the Ascotán and Carcote Salt Pans. At least two mutational steps differentiated these two haplogroups.

In the case of microsatellite analysis, the locus Tchu2422 was excluded due to the presence of null alleles, resulting in seven polymorphic loci for statistical analyses (Table S2). No evidence of stuttering or large allele dropout was found. No significant evidence for LD was found between microsatellite loci across localities. PCA using microsatellite markers (Figure 2B) revealed a clear segregation between the samples of Amincha and Ascotán–Carcote along principal component 1 (PC1), which was supported by F_ST_ values based on CR and microsatellites (Figure S1). Instead, there was a slight differentiation between Ascotán and Carcote S1 individuals along PC2. In general, pairwise F_ST_ values were higher among localities from different basins than among localities from the same basin (e.g., the Ascotán basin).

Low genetic diversity, as suggested by the scarce haplotypic diversity and reduced H_E_ values (Table S3), was found in all sampled localities. Only one haplotype was found in most of the localities of the Ascotán and Carcote Salt Pans. Amincha showed higher haplotype diversity, nucleotide diversity and H_E_ than the Salt Pan localities. This pattern was also observed when grouping all the localities of the Ascotán Salt Pan (H = 0.098; Π = 0.00011; H_E_ = 0.2723).

### 3.2. Morphometric Analyses

**Figure 3:**
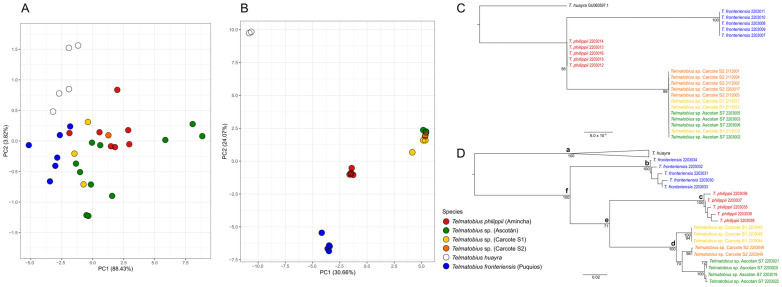
Results of the multivariate and phylogenetic analyses. (**A**) Scatter plot of PC1 to PC2 of PCA obtained using morphometric data; (**B**) scatter plot of PC1 to PC2 of PCA obtained using SNP data; (**C**), maximum likelihood tree recovered using Cytb; (**D**) maximum likelihood tree using SNP data. Within (**D**), the letters a–f indicate the clades considered in Table 3 (BFD analysis). The colours in the scatter plots and ML trees indicate specimens sampled from the same geographical unit.

The PCA results (Figure 3A) showed that *T. huayra* and *T. fronteriensis* were slightly differentiated from the other localities (i.e., Amincha, Ascotán, and Carcote). *Telmatobius fronteriensis* had lower PC1 (88.43%) values, whereas *T. huayra* had higher PC2 values than the other species. *Telmatobius philippii* individuals from Amincha were grouped along with the *Telmatobius* individuals from Carcote and Ascotán, displaying similar PC1 and PC2 values. Factor loadings (Table S4) suggest that PC1 mainly represented multivariate body size, whereas PC2 was highly correlated to head (IND, EPD) and limb variables (TaL).

PERMANOVA detected significant statistical differences (*F* = 54.43; *p* = 0.0017) among the groups. Pairwise comparisons (Table 2) revealed no significant differences (*p* > 0.05) between *T. philippii* from Amincha and *Telmatobius* sp. from Ascotán and Carcote, but they significantly differed from *T. huayra* and *T. fronteriensis*.

**Table 3:** Bayes factor delimitation analysis results. Species delimitation hypotheses with their respective ML estimation, Bayes factor (BF), and hypothesis rank are present. High BF values (BF > 10) suggest definitive support for the “splitter” hypothesis.

Species Delimitation Hypothesis	SNP Clades *	Species	ML Estimate	BF	Rank
Actual taxonomy	a,b,e	3	−9246.012	0.000	2
Splitter hypothesis	a,b,c,d	4	−8318.539	−1854.946	1
Lumper hypothesis	a,f	2	−10,140.922	+1789.820	3

* Clades are referred to the ML tree in Figure 3D.

### 3.3. Species Delimitation Analyses

The results of PCoA using SNPs (Figure 3B) revealed a well-defined differentiation among all of the units, excluding Ascotán and Carcote samples, which had similar PCoA (PC1 and PC2) values.

The topologies of the ML trees inferred using the mitochondrial (Figure 3C; final ML optimization likelihood = −1340.515067) and SNP data (Figure 3D; final ML optimization likelihood = −5176.9280) are concordant with previous studies (e.g., [25]), but with a better resolution on terminal branches in the case of the ML tree inferred using the SNP data. In both cases, *T. fronteriensis* diverge from the other units with high bootstrap values. The *Telmatobius* specimens of Ascotán and Carcote were found to be more closely related to *T. philippii* specimens than to specimens of the other studied species; however, this grouping (node d) was slightly supported (bootstrap support = 71) only in the SNP phylogeny.

The ML solution of the bPTP-ML species delimitation analysis suggested three species (Figure S2), with the *Telmatobius* localities of Ascotán and Carcote belonging to *T. philippi*. However, *T. fronteriensis* and *T. philippi* (including Ascotán and Carcote localities) partitions had low support (0.198 and 0.006, respectively).

**Figure 4:**
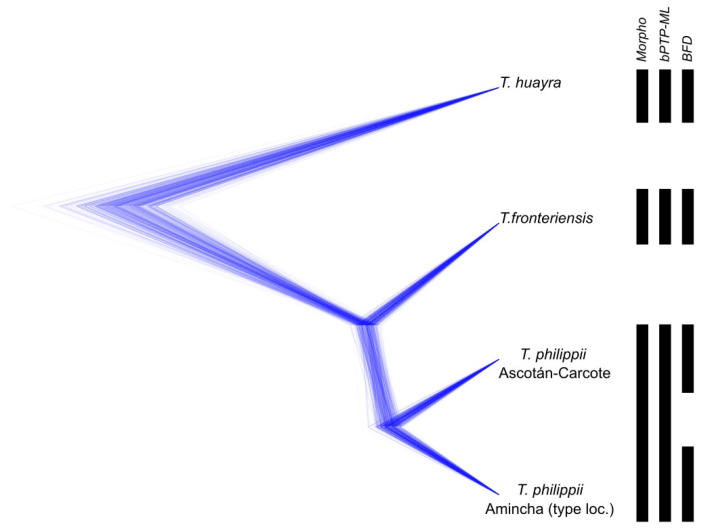
Summary scheme of the species delimitation results. The Densitree at the left represents 9000 species trees of the “splitter hypothesis” (considering Theta = 1) obtained in the path sampling analysis in BEAST2.

The BFD analysis favoured the “splitter hypothesis”, which considers the *Telmatobius* sp. of Ascotán and Carcote as a new species, over the two other species delimitation hypotheses regarding their ML estimate (Table 3). The BF difference between the splitter hypothesis and the other two species delimitation hypotheses suggest decisive support in favour of this hypothesis. A summary of the species delimitation results is depicted in Figure 4.

## 4. Discussion

### 4.1. Species Delimitation

All analyses identified *T. huayra* and *T. fronteriensis* as morphologically and phylogenetically divergent from the other studied species. In contrast, the *Telmatobius* specimens of Ascotán and Carcote were found to be closer, but not identical, to the population of the type locality of *T. philippii*, Amincha. There is disagreement among some of the results regarding these populations. On the one hand, BFD analysis favoured the “splitter hypothesis” in which the *Telmatobius* sp. of Ascotán and Carcote was considered a new species. However, morphometric and bPTP-ML results suggest that they belong to *T. philippii*. Thus, the question is should these two units be considered one or two different species?

**Figure 5:**
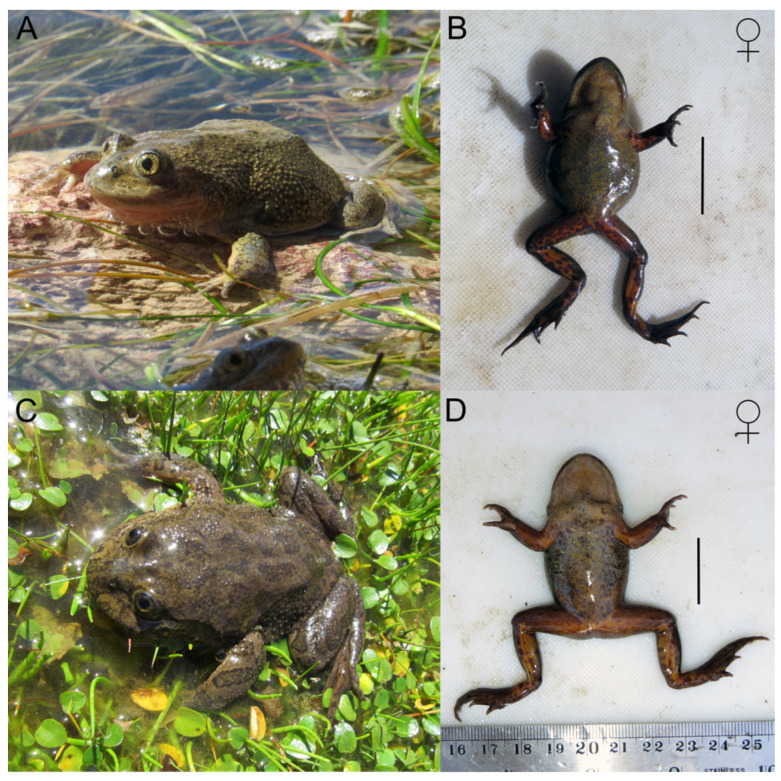
Morphological variation in *T. philippii*. (**A**,**B**) Female *T. philippii* specimen of Ascotán. (**B**–**D**) Female *T. philippii* specimen of Amincha. The bar at the right of each specimen has a length of 20 mm.

Although cryptic speciation has been suggested in Altiplanic *Telmatobius* species [29], in this case, the absence of significant morphometric differences is consistent with the minor mitochondrial differentiation (two mutational steps in CR) found between the haplogroups of *T. philippii* and *Telmatobius* sp. of Ascotán and Carcote, suggesting that they correspond to different populations of the same species rather than different species. It has been suggested that the “multispecies” coalescent model, which underlies the BFD approach used in this study, can confound the genetic structure associated with species from that of populations within species [16], which would explain why BFD analysis favoured the “splitter hypothesis”. Based on the evidence presented, we suggest that the *Telmatobius* populations of Ascotán and Carcote correspond to *T. philippii*. According to this, *T. philippii* would be present both in its type locality, Amincha, and in the salt pans of Ascotán and Carcote, corroborating previous works suggesting that the *Telmatobius* species from around Ollagüe correspond to *T. philippii* (e.g., [36,38,71]), extending the distribution of this species. In 2015, this species was classified as critically endangered mainly because of the disappearance of one of the two originally known localities (caused by the canalization of the stream in which it lived), and the remaining population in Amincha was restricted to an area of less than 10 km^2^ [72]. The *Telmatobius* localities of Ascotán and Carcote, now assigned to *T. philippii*, inhabit isolated small springs, with occupation areas of 0.01 and 0.02 km^2^, respectively [36]. Although the assignment of this population to *T. philippii* expands the recognized range of that species somewhat, the distribution of *T. philippii* is still highly restricted. From our analyses, it must be noted that the *T. philippii* localities of Carcote and Ascotán constitute a phylogeographic unit differentiated from the Amincha (type locality) unit. Because these two units comprise allopatric haplogroups and show divergence at nuclear loci, they can be considered different evolutionarily significant units (ESUs) [73] of *T. philippii*. Despite the morphometric similarity found between the two ESUs of *T. philippi*, we frequently observed females with a globose abdomen in Ascotán and Carcote, a trait that was not observed in the Amincha (type locality) specimens (Figure 5A–D) and distinguishes lacustrine *Telmatobius* [74].

### 4.2. Population Structure

The microsatellite and SNP results suggest that the populations of *T. philippii* are structured by basin limits, in which each basin (Ascotán, Carcote, and Ollagüe) possesses differentiated genotypes that are closer intra-basin than to inter-basin. A genetic structure related to basin limits has been observed previously in other Andean vertebrate species that inhabit in the Atacama Desert area, either in water frogs (e.g., *Telmatobius pefauri* [42]) or fishes (e.g., genus *Orestias* [23]). In the case of *T. philippii*, the population genetic structure appears unrelated to the geographic distance when populations from different basins are compared: the population of the *T. philippii* of Amincha is geographically closer to the Carcote Salt Pan (linear distance to spring 1 = 11 km approx.) than to the Ascotán Salt Pan (linear distance to spring 2 = 35 km approx.), but the results illustrate that the specimens of these last two basins are closely related, possessing the same predominant haplotype. This pattern matches the altitudinal differences between both Ascotán and Carcote Salt Pans, which are at a similar altitudinal range (3710–3740 m.a.s.l.), and Amincha, which is located at a higher altitude (3800–4100 m.a.s.l. approximately) than the salt pan populations. Meanwhile, past changes in the water budget, which originated and fragmented immense paleolakes during the Quaternary, could offer an alternative explanation for the differentiation pattern found in *T. philippii*. Unlike other *Telmatobius* species in Chile, the distribution of *T. philippii* falls within the maximum extent of the Tauca paleolake. This paleolake resulted from notable increases in precipitation (water budget) in the Altiplano during the Heinrich Stadial 1 (18.5 to 14.5 thousand years [ka] before present [BP]), and it is considered the most significant paleolake expansion in the Altiplano in the last 130 ka. The Tauca reached a depth of 120 m and covered an extent of 52,000 km^2^ during its highstand (16.5 to 14.5 ka BP), reaching some Chilean closed (endorheic) basins located in the western Andean–Altiplano margin, such as the Carcote Salt Pan basin [75,76,77,78]. Considering the strictly aquatic habits of *Telmatobius* species, the results (the same haplotype in Carcote and Ascotán and low divergence between the two units) strongly suggest prior hydrological connectivity among the three basins. A plausible biogeographic scenario is that during the Tauca cycle (maximum level, 3770 m a.s.l. [76]), the three basins were hydrologically connected to the rest of the paleolake, and subsequently, the geographical isolation of these systems occurred as the water level decreased because of the arid conditions in the Holocene. The Amincha ravine population of *T. philippii* would have first been isolated from the Salt Pans given its marginal position and higher altitude (3800–4100 m.a.s.l. approx.), after which Ascotán and Carcote populations would have diverged at a similar altitudinal range. Other groups of freshwater animals with populations in the Ascotán and Carcote Salt Pans, namely the gastropod genera *Biomphalaria* and *Heleobia*, and the killifish genus *Orestias*, all have divergent species [23,79,80], which is different from what was found for *T. philippii*, for which the populations of both salt pans comprised the same haplogroup, suggesting that the pattern of differentiation between these hydrographic systems can be idiosyncratic by taxa.

## 5. Conclusions

The integrative approach presented here supports the species status of *T. philippii* and *T. fronteriensis*. Despite the clear nuclear SNP differentiation between the *Telmatobius* populations of Ascotán, Carcote and the type locality of *T. philippii*, the minor mitochondrial differences between them and their conserved morphology suggest that they correspond to a new ESU of this species, increasing its geographical distribution. This highlights the importance of integrating different lines of evidence to contrast species hypotheses for poorly known endangered groups. The patterns of genetic differentiation and distribution of the Chilean species of the *Telmatobius hintoni* group would have been influenced by the expansion and regression of the Altiplano Tauca paleolake.

## Figures and Tables

**Table 1:** Geographical information of sample sites specifying the sample sizes by locality and marker. CR: mitochondrial control region; Cytb: cytochrome-b; SSR: simple sequence repeat (microsatellite); SNP: single-nucleotide polymorphism. Specimens used in morphological analyses are specified in Appendix A.

Species	Locality	Geographical Coordinates	Altitude (m.a.s.l.)	n CR	n SSR	n Cytb	n SNP
*T. fronteriensis*	Puquios	21°00′02.49″ S 68°23′08.80″ W	4167	-	-	5	5
*T. philippii*	Amincha	21°11′07.25″ S 68°21′30.00″ W	4067	21	24	5	5
*Telmatobius* sp.	Carcote spring 1	21°16′58.60″ S 68°19′28.00″ W	3709	14	12	3	3
*Telmatobius* sp.	Carcote spring 2	21°17′43.59″ S 68°20′09.13″ W	3702	1	1	5	2
*Telmatobius* sp.	Ascotán spring 2	21°29′21.20″ S 68°15′24.80″ W	3732	8	10	-	-
*Telmatobius* sp.	Ascotán spring 3	21°29′27.80″ S 68°15′25.60″ W	3734	8	10	-	-
*Telmatobius* sp.	Ascotán spring 5	21°29′47.20″ S 68°15′24.50″ W	3729	12	10	-	-
*Telmatobius* sp.	Ascotán spring 6	21°29′52.80″ S 68°15′24.40″ W	3732	12	10	-	-
*Telmatobius* sp.	Ascotán spring 7	21°32′03.11″ S 68°15′50.98″ W	3728	9	10	4	4
*Telmatobius* sp.	Ascotán spring 11	21°41′13.90″ S 68°12′54.00″ W	3740	9	7	-	-
*Telmatobius* sp.	Ascotán spring 12	21°35′13.90″ S 68°14′32.30″ W	3735	2	1	-	-
*T. huayra*	Sol de Mañana, Bolivia	22°7′44.70″ S 67°16′33.80″ W	4188	-	-	1	2
Total				96	95	23	21

**Table 2:** Post hoc morphometric comparisons. The pairwise permutation test results include FDR nonadjusted and adjusted *p*-values. Significant comparisons (*p* < 0.05) are indicated in bold.

Species 1	Species 2	*p*-Value	FDR-Adjusted *p*
*Telmatobius* sp. Ascotán	*T. philippii*	0.5076	0.5076
*Telmatobius* sp. Carcote	*T. philippii*	0.0817	0.1021
*T. huayra*	*T. philippii*	**0.0046**	**0.0167**
*T. fronteriensis*	*T. philippii*	**0.0026**	**0.0167**
*Telmatobius* sp. Carcote	*Telmatobius* sp. Ascotán	0.2970	0.3300
*T. huayra*	*Telmatobius* sp. Ascotán	**0.0183**	**0.0305**
*T. fronteriensis*	*Telmatobius* sp. Ascotán	**0.0074**	**0.0185**
*T. huayra*	*Telmatobius* sp. Carcote	**0.0160**	**0.0305**
*T. fronteriensis*	*Telmatobius* sp. Carcote	**0.0050**	**0.0167**
*T. fronteriensis*	*T. huayra*	**0.0267**	**0.0381**

## Data Availability

Sequence data are available in the Genbank repository (Genbank Accession Numbers PQ626324-PQ626419). Microsatellite and SNP data matrices analysed during the current study are available in the datos.uchile.cl repository: https://doi.org/10.34691/UCHILE/PBCOJA.

## Supplementary Materials

The following supporting information can be downloaded at: https://www.mdpi.com/article/10.3390/ani14243612/s1, Figure S1: Population differentiation in *T. philippii* per sampling locations. Heatmaps depicting pairwise F_ST_ values among localities based on CR and microsatellite data. Statistically significant comparisons (*p* < 0.05) are indicated by an asterisk. Carcote S2 sample was grouped within Carcote S1 due to small sample size (N = 1). Localities were ordered geographically from north to south. Figure S2: Results of the bPTP-ML analysis for species delimitation using Cytb data. Table S1: Sample sizes per locality and life stage. A, adult; J, juvenile; L, larvae. Table S2: Primer sequences and characteristics for ten microsatellite loci for *T. philippii*. Table S3: Genetic diversity in *T. philippii* based on the analysis of CR and microsatellites. N, sample size per locality; S, number of polymorphic sites; K, number of haplotypes; H, haplotype diversity; Π, nucleotide diversity; Pairw. diffs., average number of pairwise differences; H_E_, expected heterozygosity; H_O_, observed heterozygosity; N_A_, average number of alleles per locus; F_IS_, inbreeding coefficient. H, Π, H_E_ and H_O_ are expressed as mean values ± standard deviation. Statistically significant F_IS_ values (*p* < 0.05) are indicated by an asterisk. Table S4: Factor loadings obtained in principal component analysis. Variable abbreviations correspond to those indicated in methodology.

## Appendix A

Collection codes of the specimens measured for the morphometric analyses. DBGUCH: Herpetological Collection of the Departamento de Biología Celular y Genética of the Universidad de Chile; CBF: Bolivian Collection of Fauna.

*Telmatobius philippii*: Salar de Ascotán, Región de Antofagasta, Chile: DBGUCH: 604016, 604002, 604017, 1109001, 809055, 809054, 505011, 1211024, 1304001, 1309006, 1501026; Salar de Carcote, Región de Antofagasta, Chile: DBGUCH 1211034, 1309001, 1309002, 1309003; Quebrada Amincha, Región de Antofagasta, Chile: 1110055, 1110054, 1211037, 1309017, 1309016, 1501023, 1309015.

*Telmatobius fronteriensis*: Puquios, Región de Antofagasta, Chile: DBGUCH 1304006, 1304007, 1309009, 1501005, 1501001, 1501009.

*Telmatobius huayra*: Campamento Khastor, Provincia Sud-Lípez, Departamento de Potosí, Bolivia: CBF 1221, 1222, 1223 (holotype); Departamento de Potosí, Bolivia: CBF 6281, 6283.
