# Supplementary material for: Delimitation of Endangered Telmatobius Species (Anura: Telmatobiidae) of the Chilean Salt Puna
Source: Animals (Basel). 2024 Dec 15;14(24):3612. doi: 10.3390/ani14243612 (PMC11672803; doi:10.3390/ani14243612)
Supplement: Supplementary file 1 [file animals-14-03612-s001.zip › animals-3282336-supplementary.pdf]

Supplementary Materials

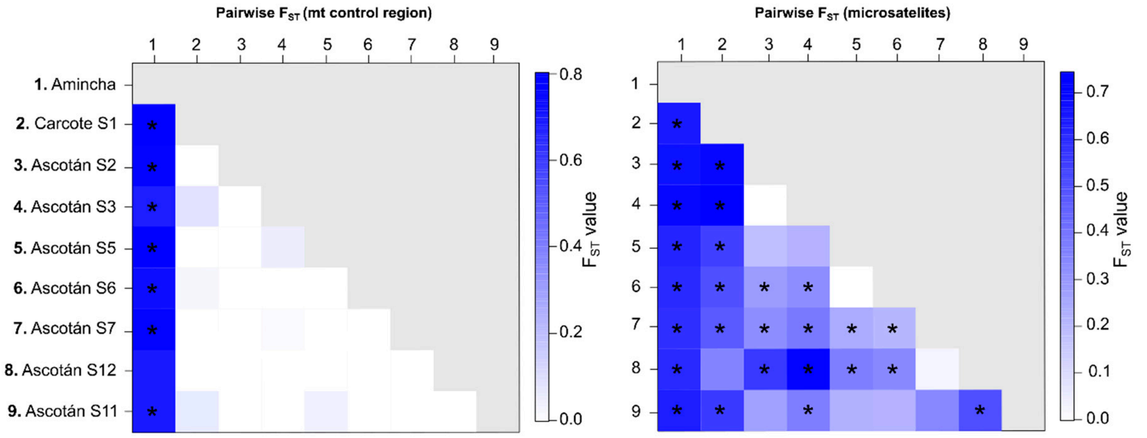

Figure S1. Population differentiation in *T. philippii* per sampling locations. Heatmaps depicting pairwise F_{ST} values among localities based on CR and microsatellite data. Statistically significant comparisons (P<0.05) are indicated by an asterisk. Carcote S2 sample was grouped within Carcote S1 due to small sample size (N=1). Localities were ordered geographically from north to south.

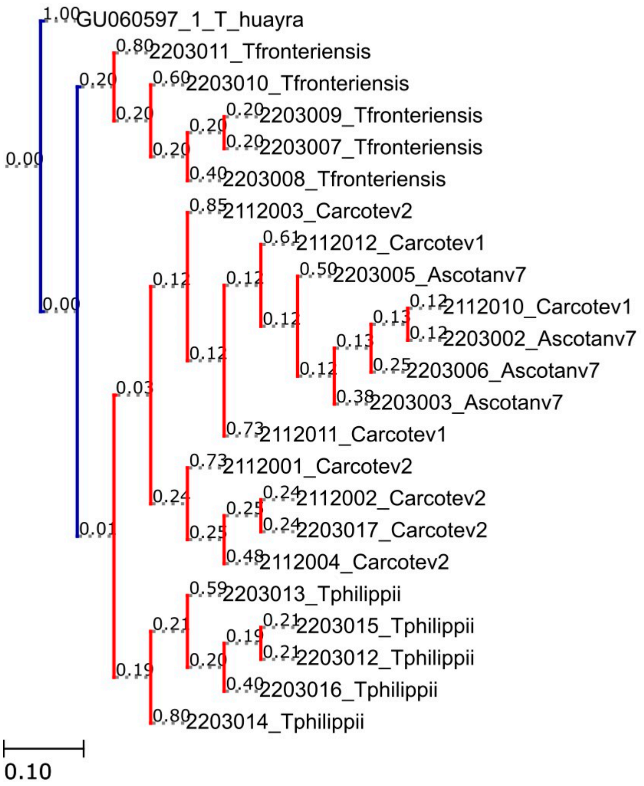

Figure S2. Results of the bPTP-ML analysis for species delimitation using Cytb data.

Table S1. Sample sizes per locality and life stage. A, adult; J, juvenile; L, larvae.

Species	Locality	n CR	n SSR	n Cytb	n SNP
T. fronteriensis	Puquios	-	-	5 (5A)	5 (5L)
T. philippii	Amincha	21 (5A,1J,15L)	24 (3A,2J,19L)	5 (5A)	5 (5A)
Telmatobius sp.	Carcote spring 1	14 (1A,1J,12L)	12 (2A,1J,9L))	3 (3L)	3 (3L)
Telmatobius sp.	Carcote spring 2	1 (1A)	1 (1A)	5 (5A)	2 (2A)
Telmatobius sp.	Ascotán spring 2	8 (8L)	10 (10L)	-	-
Telmatobius sp.	Ascotán spring 3	8 (8L)	10 (10L)	-	-
Telmatobius sp.	Ascotán spring 5	12 (12L)	10 (10L)	-	-
Telmatobius sp.	Ascotán spring 6	12 (12L)	10 (1A,9L)	-	-
Telmatobius sp.	Ascotán spring 7	9 (3A,3J,3L)	10 (2A,3J,5L)	4 (4A)	4 (4A)
Telmatobius sp.	Ascotán spring 11	9 (5A,4J)	7 (4A,3J)	-	-
Telmatobius sp.	Ascotán spring 12	2 (1A,1L)	1(1A)	-	-
T. huayra	Sol de Mañana, Bolivia	-	-	1*	2 (2A)
Total		96 (16A,9J,71)	95 (14A,9J,72L)	23 (19A,3L,1*)	21 (13A,8L)

*data from Genbank.

Table S2. Primer sequences and characteristics for ten microsatellite loci for *T. philippii*.

Locus name	Motif	Primer forward	Primer reverse	Fluorophore	T_a (C°)	Alleles range (pb)	Number of alleles observed
T_2422	$(AGT)_{10}$	GGTAAGTATTTACATGATCG	TAGTACTACTGCCACTACTGT	NED	58	175-204	2
T_1463	$(GATA)_{13}$	CTACTTAAATAGTGACTGCC	GTATATGTAGGGTTCGACT	PET	56	118-138	6
Tchus_21*	$(TAGA)_{13}$	GTACTTGATTTTATGCAACT	CTTTATTTTCTTACCCAAG	VIC	56	228-244	5
Tchus_22*	$(AGAC)_{9}$	TACACAAACAAATCTAGTCTC	GAATCTGCTATTGTAGAGTAT	FAM-6	58	176-184	3
Tchus_19*	$(TCT)_{5}$	AAATCTGAAATTAGACTCCT	GTCACAGAGACAAGTATCG	FAM-6	58	170-233	7
Tchus_17*	$(ATC)_{7}$	AGTTGACTATGGTTAAAGTG	ATATGCAGCTATACATACAGT	PET	56	132-138	3
T_588	$(AGAT)_{7}$	CAGGGTTATATTTTACAGTAG	ATACAGTGAAGTTCTACCAC	NED	56	272-284	4
Tchus_7*	$(TAA)_{5}$	CTGTGTTTGATTATTAGTTG	GTCTAATAGTCTTTCACTTTG	VIC	56	258-261	2

*Markers obtained from Fabres *et al*. (2019).

Table S3. Genetic diversity in *T. philippii* based on the analysis of CR and microsatellites. n, sample size per locality; S, number of polymorphic sites; K, number of haplotypes; H, haplotype diversity; Π, nucleotide diversity; Pairw. diffs., average number of pairwise differences; H_E, expected heterozygosity; H_O, observed heterozygosity; N_A, average number of alleles per locus; F_{IS}, inbreeding coefficient. H, Π, H_E and H_O are expressed as mean values ± standard deviation. Statistically significant F_{IS} values ($P<0.05$) are indicated by an asterisk.

Sampling site	n_{mt}	S	K	H	Π	Pairw. diffs.	n_{ssr}	H_E	H_O	N_A	F_{IS}
Amincha	21	3	4	0.348 ±0.128	0,00041 ±0.00016	0.37143	24	0.3511 ±0.2738	0.3526 ±0.3313	2.8571	0.01784
Carcote V1	14	0	1	0.000	0.00000	0.00000	12	0.0536 ±0.1417	0.0238 ±0.0630	1.1429	0.58491
Carcote V2	1	0	1	-	-	-	1	-	-	1	-
Ascotán V2	8	0	1	0.000	0.00000	0.00000	10	0.1472 ±0.1318	0.1321 ±0.1248	2.0000	0.16030
Ascotán V3	8	1	2	0.250 ±0.180	0.00028 ±0.00020	0.25000	10	0.0996 ±0.1319	0.1175 ±0.1596	1.4286	-0.12548
Ascotán V5	12	0	1	0.000	0.00000	0.00000	10	0.2214 ±0.2489	0.1714 ±0.2215	1.5714	0.27517*
Ascotán V6	12	1	2	0.167 ±0.134	0.00018 ±0.00015	0.16667	10	0.2585 ±0.2199	0.2746 ±0.2682	1.8571	-0.00801
Ascotán V7	9	0	1	0.000	0.00000	0.00000	10	0.2801 ±0.2720	0.2603 ±0.2693	1.8571	0.12417
Ascotán V11	9	1	2	0.222 ±0.166	0.00025 ±0.00019	0.22222	7	0.2049 ±0.2561	0.2313 ±0.3091	1.4286	-0.04615
Ascotán V12	2	0	1	0.000	0.00000	0.00000	1	-	-	1	-
All	96	7	7	0.409 ±0.055	0.00097 ±0.00014	0.83575	95	0.5043 ±0.1250	0.2127 ±0.1775	4.2857	0.58186*

Table S4. Factor loadings obtained in principal component analysis. Variable abbreviations correspond to those indicated in methodology.

Variable	PC1 loadings	PC2 loadings
Snout to vent length (SVL)	0.3120	0.0138
Head width (HW)	0.3109	-0.1934
Head length (HLt)	0.2882	0.1529
Femur length (FmL)	0.3049	0.2486
Tibia length (TbL)	0.3089	0.2963
Internostrils distance (IND)	0.2963	-0.3962
Eye-snout distance (ES)	0.2992	-0.2993
Distance between anterior eye commissures (EAD)	0.3086	-0.2178
Distance between posterior eye commissures (EPD)	0.2899	-0.4034
Tarsus length (TrL)	0.2961	0.4949
Length of the fourth toe (T4L)	0.3004	0.2959
